# The role of WhatsApp® in medical education; a scoping review and instructional design model

**DOI:** 10.1186/s12909-019-1706-8

**Published:** 2019-07-25

**Authors:** E. Coleman, E. O’Connor

**Affiliations:** Department of Intensive Care and Anaesthesia, St James’s Hospital, Trinity College, Dublin 8, Dublin, Ireland

**Keywords:** eLearning, mLearning, Instant messenger applications, Blended learning, Social media learning, Learning theory

## Abstract

**Background:**

Technological advances have driven huge change in educational practices though concerns exist about a lack of evidence informing this change, in particular with social media-based medical education activities. The purpose of this study was to conduct a scoping review of WhatsApp use in medical education, narratively describing how it has been used and evaluated, and the theoretical considerations in relevant articles.

**Methods:**

A modified 5-stage scoping review model was used. We performed 2 searches from February 2009 to February 2019 in EBSCO, SCOPUS, Web of Science, EMBASE, Medline PubMed and Google Scholar) using the term “WhatsApp” in all search fields. A 3-stage process for study selection was performed. Only original articles in English presenting original data about WhatsApp in medical education were included. The Kirkpatrick model of training evaluation was used to describe learning outcomes in included studies.

**Results:**

Twenty-three articles were selected for review. Three strategies for WhatsApp use were apparent; primarily educational use with a pre-defined curriculum (*n* = 5), primarily educational use without a curriculum (*n* = 11), and primarily non-educational use (*n* = 7). Most of the educational studies used an online moderator and were in a local hospital or university department. Studies not primarily educational were national or international and seldom included an online moderator. All 5 studies with a pre-defined curriculum reported Kirkpatrick level 2 learner knowledge outcomes. A majority of the remaining studies only reported Kirkpatrick level 1 learner attitudes. Seven studies with 647 participants reported an improvement in learners’ knowledge following WhatsApp learning, though methodological weaknesses were apparent. Evidence for underlying learning theory considerations were scant throughout the studies.

**Conclusions:**

WhatsApp is popular and convenient in medical education. Current published literature suggests it may also be effective as a medical learning tool. By combining the 3 strategies for WhatsApp use and the exploration-enactment-assessment integrated learning design framework, we propose an instant messenger design model for medical education. This may address the need for theory-driven instructional design in social media learning. Further research would clarify the role of WhatsApp and our design model in this area.

## Background

Advances in information technology have driven huge changes in many aspects of human behaviour and communication. These changes have had considerable implications for educational practices. In particular, the last decade has seen widespread access to mobile internet devices (MIDs) which in turn have expanded educational opportunities outside the classroom setting [1]. Learners with a suitable MID and a link to the world wide web have ready access to a wide range of multimedia learning resources, collectively known as mobile learning (mLearning) [2].

MIDs enable access to two main resources for the medical learner; applications and social media (SM) networks. The former, an extensive list, include UpToDate®, Medscape®, peer-reviewed journals and numerous podcasts [3]. The latter includes wikis, online blogs, YouTube®, and instant messenger applications (IMAs) such as Facebook®, WhatsApp®, Twitter® and WeChat® [4]. IMAs, while not primarily educational in nature, share common features which can facilitate learning; group collaboration, peer communication independent of time and geographical location, and multimedia message sharing [5–7].

WhatsApp®, a free standalone IMA launched in 2009, has over 1 billion active users in 180 countries [8]. In December 2017, it was the most popular IMA in South America, India, Russia, Eastern Europe, the UK and Africa, and the second most popular in North America [9]. As a secure educational tool it uses two-way opt-in for all users, allows the monitoring of users’ activity and message reading, and has end-to-end encryption [10]. It has some theoretical benefits over other IMAs; prior registration with a SM network is not required, and it is more favourable if internet bandwidth or speeds are poor [10].

The use of SM and IMAs as learning tools has met with resistance from some medical faculty members. While this in part relates to technical unfamiliarity, real concerns exist about professional implications of SM use [11] and the quality of evidence supporting their learning benefits [12]. One recent review of SM in medical education highlighted how the 13 included studies tended “to focus on evaluating the effective outcomes … as opposed to understanding any linkages between social media and performance outcomes”(p369) [13]. A more recent larger postgraduate education review drew similar conclusions [14]. A large majority of studies in these reviews evaluated Facebook® but contained little information about other media or IMAs.

A key concern therefore is that the advance of SM and IMA learning in medical education may be driven more by social behaviour and the high availability and low cost of technology rather than by empirical educational research or by theory-driven instructional design. What is the evidence that recent technology advances, and the learning that they have promoted, have brought about improvements in educational outcomes? Furthermore, if such evidence exists, does it have a sound basis in the principles of educational theory?

Accordingly, the objective of this study was to explore published literature, using a scoping review framework, to evaluate the role of WhatsApp®, a ubiquitous instant messaging application, as a medical learning tool, and to articulate the extent to which this literature has a foundation in educational theory.

## Methods

We used a modified 5-stage model for scoping reviews proposed by Arksey and O’Malley [15, 16]. These stages are (i) identifying research questions, (ii) identifying relevant articles, (iii) study selection, (iv) charting the data and (v) collating, summarising, and reporting the results. The purposes of the review were to define the nature of existing research into WhatsApp® for medical learning and to identify a focus for future research. In keeping with scoping review guidelines, we provided a description of each study but did not apply a quality assessment tool to each [16].

### Identifying the research questions

The selected research questions were: (1) How has WhatsApp® been used as a learning tool in medical education? (2) How has WhatsApp® been evaluated as a learning tool in medical education? (3) What educational theoretical principles were evident in studies of WhatsApp® as a learning tool in medical education?

### Identifying relevant studies

The first literature search was performed across six databases (EBSCO, SCOPUS, Web of Science, EMBASE, Medline, and Google Scholar) from February 2009, when WhatsApp® was created, until July 2018. During manuscript rewriting, in February 2019, a second search across the same databases was performed. We used the search term “WhatsApp” applied to the text, title and abstract of all publications. Reference lists from included studies were also searched. Search results were collected, organized and shared between authors using Mendeley Reference Manager®.

Relevant studies were identified using a three stage process, which involved title and abstract screening, review of abstracts, and full-text review. The first 2 stages were done independently by each author and the final stage was done collectively by both authors. Article relevance was judged by the following criteria; (i) original articles, (ii) published in English, (iii) presenting unique data (original data presented in the study) (iv) describing the use of WhatsApp® as an educational tool in a medical setting.

### Selecting studies for inclusion

**Fig. 1:**
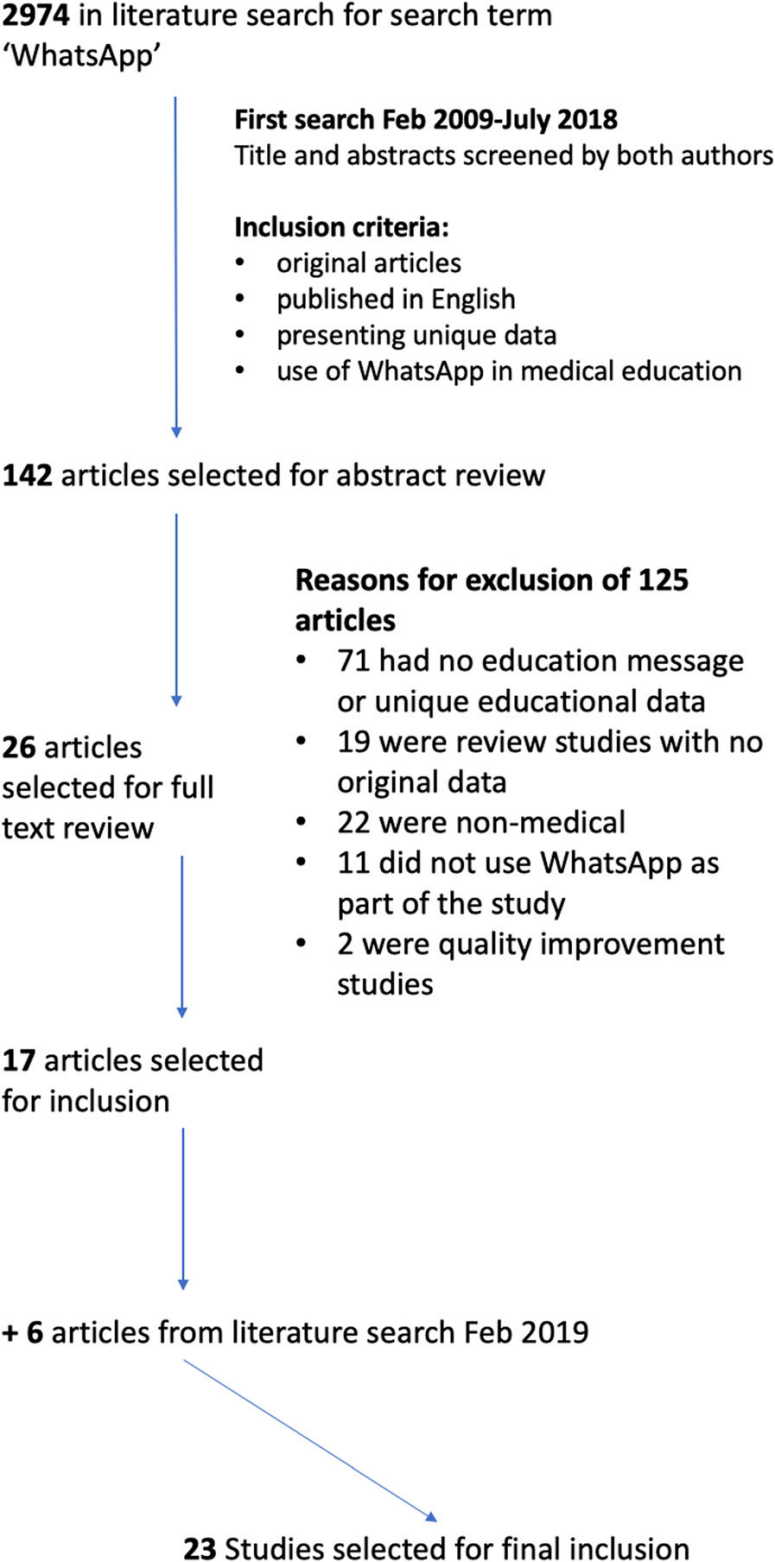
Study search strategy and reasons for study exclusions

A total of 2974 articles were identified on the first search from which 23 article were selected for review. Details of study inclusions and exclusions are shown in Fig. 1.

### Charting the data

Appropriate study data were condensed in tabulated form for each study. Each author performed this step independently for all articles and a final table was compiled following collaborative discussion between the authors (Table 1).

**Table 1:** Twenty-three studies included in the scoping review of WhatsApp in medical education

Author Country	Study title	Journal Year Specialty Under−/Postgraduate	Study design Single arm or not Main data type(s)	Sample size Description of intervention Data collection tool(s)	Key messages from study findings	Levels of learning outcomes Articulated educational theory
Mayer et al. [17] United Kingdom	Transfusion education: can using social media help improve training? The West Midlands experience	British Journal of Haematology 2017 Haematology Postgraduate	Retrospective observational study Single arm Qualitative	*N* = 25 WA case-based transfusion tutorials for 9 months Survey	13 WA tutorials over 9 months Feedback about WA pros and cons reported Participant’s work on WA used as a basis for workplace-based assessment for that doctor	Kirkpatrick level 1 outcomes. THEORY ARTICULATED: None
Bhesania et al. [18] USA	Using social media to advance medical education in a university affiliated community residency program	Journal of General Internal Medicine 2018 Cardiology Postgraduate	Prospective observational study Single arm Quantitative	*N* = 68 ECG learning group on WA for 2 years WA discussion analysis	167 ECGs and 808 messages posted Evidence of clinical reasoning, establishing diagnoses and proposing treatment in discussions	No Kirkpatrick outcomes THEORY ARTICULATED: None
Elshaikh et al. [19] USA	WhatsApp as a supplemental learning tool for pathology	Laboratory Investigation 2018 Pathology Postgraduate	Retrospective observational study Single arm Quantitative	*N* = 24 Pathology group on WA for 2 years Survey, WA discussion analysis	230 pathology cases discussed Feedback about WA pros and cons discussed 87.5% users learned “new entities” on WA	Kirkpatrick level 1 outcomes THEORY ARTICULATED: None
Alkhalaf et al. [20] Saudi Arabia	The impact of WhatsApp use on academic achievement among Saudi medical student	Medical Teacher 2018 Medical education Undergraduate	Retrospective observational study Single arm Quantitative	*N* = 160 Correlation between end of term results and WA usage Survey	WA used by minority (26.9%) for education No association between summative GPAs and WA usage	Kirkpatrick level 1 outcomes THEORY ARTICULATED: None
Bakshi et al. [21] India	Role of WhatsApp-based discussions in improving residents’ knowledge of post-operative pain management: a pilot study	Korean Journal of Anaesthesia 2017 Anaesthesia-pain Postgraduate	Prospective cohort study Single arm Quantitative	*N* = 38 Anaesthesia/Pain WA learning group for 3 months Survey, WA discussion analysis, Pre−/post-intervention knowledge and behaviour assessment	Significant improvement in post-intervention knowledge scores (73.6% vs 69.1%, *p* = 0.031) Significant improvement in learner behaviour (documentation of epidural anaesthesia efficacy) to 3 months	Kirkpatrick level 1, 2 and 3 outcomes Level 3 outcome demonstrated 3 months after teaching THEORY ARTICULATED: None
Blumenfeld et al. [22] Israel	Real time medical learning using the WhatsApp cellular network: a cross sectional study following the experience of a division’s medical officers in the Israel Defence Forces	Disaster and Military Medicine 2016 General Medicine Postgraduate	Retrospective observational study Single arm Quantitative	*N* = 41 Peer discussion among military medical professionals on WA for 2 years WA discussion analysis	478 questions and 531 responses Categorisation of WA messages into textual/visual, questions/responses and subject matter 34% of messages related to clinical discussion	No Kirkpatrick outcomes THEORY ARTICULATED: None
Carmona et al. [10] International	Realising the potential of real-time clinical collaboration in maternal-fetal and obstetric medicine through WhatsApp	Obstetric medicine 2018 Maternal-fetal medicine Postgraduate	Retrospective observational study Single arm Quantitative study	N = 41 WA education and clinical discussion group in MFM for 2 years Survey, WA discussion analysis	534 of 5050 (10.6%) related to clinical topics; 35% had educational purpose Categorisation of messages into advice seeking, clinical case sharing, educational content, and miscellaneous content Feedback about WA pros and cons reported. 97% reported “increased knowledge in rare cases”	Kirkpatrick level 1 outcomes THEORY ARTICULATED: None
Gon et al. [23] India	Effectivity of e-learning through WhatsApp as a teaching learning tool	MVP Journal of Medical Sciences 2017 Pathology Undergraduate	Prospective randomized crossover study Qualitative and quantitative	*N* = 80 Blended pathology learning using WA for 5 months. Compared with traditional lectures Survey, Pre−/post-intervention knowledge assessment	More questions asked and answered in WA than in lectures. Both WA and lectures improved learners’ scores but no difference in improvement between teaching methods Feedback about WA pros and cons reported	Kirkpatrick levels 1 and 2 outcomes THEORY ARTICULATED: mLearning (mobile learning)
Goyal et al. [24] India	WhatsApp for teaching pathology postgraduates: a pilot study	Journal of Pathology Informatics 2017 Pathology Postgraduate	Prospective observational study Single arm Quantitative.	*N* = 69 WA pathology discussions for 4 weeks Survey, WA discussion analysis	16 pathology cases discussed Feedback about WA pros and cons reported > 1/3 of users posted no messages	Kirkpatrick level 1 outcomes THEORY ARTICULATED: None
Hayward et al. [25] United Kingdom	Virtual learning communities for faculty members: does WhatsApp work?	Medical Education 2018 Clinical education faculty Postgraduate	Prospective observational study Single arm. Qualitative or Quantitative - unclear	*N* = 58 WA discussion groups for faculty educators for 1 year Survey	Feedback about WA pros and cons reported Effective way for faculty to feel “connected to the medical school”	Kirkpatrick level 1 outcomes THEORY ARTICULATED: None
Kaliyadan et al. [26] India	What’s up dermatology? A pilot survey of the use of WhatsApp in dermatology practice and case discussion among members of WhatsApp dermatology groups?	Indian Journal of Dermatology, Venereology and Leprology 2016 Dermatology Postgraduate	Retrospective observational study Single arm Quantitative	*N* = 100 Dermatology WA case discussions. Unknown duration of discussions. Survey	Feedback about WA pros and cons reported 54% of users thought photo image quality suboptimal 70.5% of users in more than one WA group	Kirkpatrick level 1 outcomes THEORY ARTICULATED: None
Khan et al. [27] Saudi Arabia	Impact of network aided platforms as educational tools on academic performance and attitude of pharmacology students	Pakistan Journal of Medical Science 2017 Pharmacology Undergraduate	Prospective cluster randomisation study Quantitative	*N* = 72 in 6 universities Blended learning study for 5 months comparing lectures, WA/lectures (W/L) and Learning management system/WA/lectures. (L/W/L) End of term summative assessments	Knowledge outcomes significantly higher in W/L and L/W/L than lectures but no difference between W/L and L/W/L	Kirkpatrick level 2 outcomes THEORY ARTICULATED: mLearning, eLearning
Loo et al. [28] Malaysia	Use of WhatsApp in assisting psychiatry learning	Medical Education 2016 Psychiatry Postgraduate	Retrospective observational study Single arm Qualitative or Quantitative - unclear	*N* = 122 WA discussion group to support psychiatry exam preparation. Unknown duration of discussions WA discussion analysis	Feedback about WA pros and cons reported Applicability to “countries with limited resources”	Kirkpatrick level 1 outcomes THEORY ARTICULATED: Peer-to-peer learning
Mazzuoccolo et al. [29] Argentina	WhatsApp: a real-time tool to reduce the knowledge gap and share the best clinical practices in psoriasis	Telemedicine Journal and e-Health 2019 Dermatology Postgraduate	Prospective observational study Single arm study Quantitative	*N* = 80 WA discussion group for 1 year to link dermatologists Survey and WA discussion analysis	197 dermatology questions posted, all answered in discussion Impact of WA discussions on participants’ clinical practice and learning reported	Kirkpatrick level 1 outcomes THEORY ARTICULATED: None
Bukhari et al. [30] Canada	Enhancing internal medicine trainees’ nephrology competency: Queen’s Nephrology e-learning using WhatsApp study	Internal Medicine 2017 Nephrology Postgraduate	Prospective observational study. Pre−/post-intervention single arm Quantitative	*N* = 27 WA discussion on nephrology topics for 16 weeks Survey of self-reported confidence in medical knowledge	Self-reported increase in confidence in diagnosing and managing nephrology conditions Early termination due to trainee non-participation	Kirkpatrick level 1 outcomes. THEORY ARTICULATED: None
Raiman et al. [31] United Kingdom	WhatsApp messenger as a tool to supplement medical education for medical students on clinical attachment	BMC Medical Education 2017 Internal Medicine Undergraduate	Prospective descriptive study Single arm Qualitative and quantitative	*N* = 19 Blended learning using WA discussions with face-to-face problem-based learning WA discussion analysis, Structured interviews	WA content analysis: a) organizational b) educational c) social Emergent themes on WA usage: a) ease of use b) fosters understanding c) sharing resources electronically d) accessing recorded discussions e) generating other learning opportunities f) intrusiveness g) lack of face-to-face interaction	Kirkpatrick level 1 outcomes THEORY ARTICULATED: mLearning
Khanna et al. [32] Uncertain	“WhatsApp”ening in orthopaedic care: a concise report from a 300-bedded tertiary care teaching centre	European Journal of Orthopaedic Surgery and Traumatology 2015 Orthopaedics Postgraduate	Prospective observational study Pre−/post-intervention single arm Quantitative	*N* = 8 WA group to share information about new orthopaedic patient admissions. Unknown duration of discussions Pre−/post-intervention knowledge assessment	Knowledge about orthopaedic diagnoses significantly improved No improvement in knowledge about orthopaedic management Feedback about WA pros and cons reported	Kirkpatrick level 2 outcomes THEORY ARTICULATED: NONE
Kochar et al. [33] USA	Disrupting fellow education through group texting. WhatsApp in fellow education?	Journal of the American College of Cardiology 2018 Cardiology Postgraduate	Prospective observational study Single arm Quantitative	*N* = 56 Cardiology WA discussion group for 5 months Survey, WA discussion analysis	“> 500 images and videos shared” in WA discussions Guidelines suggested for successful implementation of WA-based learning programme	Kirkpatrick level 1 outcomes THEORY ARTICULATED: NONE
Ranjan et al. [34] India	WhatsApp-assisted learning of anatomy as an adjuvant to traditional class-room learning: achievements and prospect	International journal of anatomy and research 2017 Anatomy Undergraduate	Prospective observational study Single arm Quantitative	*N* = 150 Blended learning combining WA anatomy discussions with standard teaching for 8 months Survey	Feedback about WA pros and cons reported WA used to ask questions about topics unclear from lectures Early inclusion of all learners in learning process “anytime and anywhere” learning	Kirkpatrick level 1 outcomes THEORY ARTICULATED: mLearning, Andragogy
Mohesh et al. [35] India	Perceptions on M-learning through WhatsApp application	Journal of education technology in health sciences 2016 Physiology Undergraduate	Prospective observational study Single arm Qualitative and quantitative	*N* = 46 Daily physiology WA topics discussed for 46 days Survey	Feedback about WA pros and cons reported Short relevant messages favoured over long messages Suited to the “smart generation”	Kirkpatrick level 1 outcomes THEORY ARTICULATED: mLearning, eLearning
Dyavarishetty et al. [36] India	An interventional study to assess the effectiveness of “WhatsApp” as a teaching learning tool in community medicine	International journal of community medicine and public health 2017 Community medicine Undergraduate	Prospective observational study Single arm Qualitative and Quantitative	*N* = 49 Blended learning with WA discussion in 4 modules “complemented existing learning” for 4 months Survey, Pre−/post-intervention knowledge assessment, WA discussion analysis, structured interviews	Knowledge improvement in 2 of 4 modules Drop in participation over course of study Feedback about WA pros and cons reported	Kirkpatrick level 1 and 2 outcomes THEORY ARTICULATED: NONE
Mohanakrishnan et al. [37] India	WhatsApp enhances medical education: is it the future?	International journal of medical science and public health 2017 Virology Undergraduate	Prospective randomized crossover study Qualitative and quantitative	*N* = 100 Blended learning comparing WA preparation for 2 days before 2 lectures with lectures alone Survey, post-intervention knowledge assessment	Flipped classroom model in intervention group Feedback about pros and cons of WA reported Significantly better knowledge scores in blended learning group than lecture group after both teaching sessions	Kirkpatrick level 1 and 2 outcomes THEORY ARTICULATED: NONE
Maske et al. [38] India	Feasibility, effectiveness, and students’ attitude toward using WhatsApp in histology teaching and learning	Journal of education and health promotion 2017 Histopathology Undergraduate	Prospective observational study Single arm Quantitative	*N* = 250 Three 2-month WA discussions about histology topics Survey, Pre−/post-intervention knowledge assessment	Significant improvement in performance between pre- and post-intervention tests for all 3 lessons Feedback about WA pros and cons reported “anytime anywhere learning”	Kirkpatrick level 1 and 2 outcomes THEORY ARTICULATED: NONE

*WA* WhatsApp, *GPA* Grade point average, mLearning: mobile learning. Kirkpatrick 1 outcomes: learner attitudes. Kirkpatrick 2 outcomes: learner knowledge or confidence. Kirkpatrick 3 outcomes: learner behaviour

### Collating, summarising and reporting the results

After data tabulation, we adopted a narrative approach to summarising and reporting the data, informed by our 3 research questions. We used consensus statements to guide the description of study design [39]. The Kirkpatrick Model of Training Evaluation was used as a framework for describing the learning outcomes in each study [40].

## Results

### Summary of the articles

Twenty-three articles were included in the review, all published in the years 2015–2018 [10, 17–38]. Fourteen enrolled postgraduate and nine [20, 23, 27, 31, 34–38] enrolled undergraduate learners. A wide variety of subspecialties were represented across the basic health sciences [19, 23, 24, 27, 34, 35, 37, 38], clinical health sciences and in medical education [25].

Sixteen (69.6%) of the twenty-three studies had a prospective design. Three used random allocation of participants to WhatsApp® or control groups [23, 27, 37]. Five studies used participants as their own controls, adopting a pre−/post-intervention design [21, 30, 32, 36, 38]. The fifteen remaining studies had a single arm design, two of which collected mainly qualitative data [17, 31].

The most common study setting for the WhatsApp® group usage was locally in either a university setting [20, 23, 25, 31, 34–38] or a hospital department [17, 18, 21, 24, 30, 32, 33]. Six studies had a national setting [19, 22, 26–29]. Only one study had international WhatsApp® group participation [10].

Paradigmatically, most of the studies (15; 65.2%) adopted a positivist quantitative methodology. One study used an interpretivist approach [17] and two did not specify an overarching methodology [25, 28]. The final five studies combined qualitative and quantitative data but fell short of articulating a pragmatist paradigm or a mixed-methods design [23, 31, 35–37]. Data collection was mainly using participant surveys (18/23; 78.3%) and content analysis of WhatsApp® discussions (10/23; 43.5%). Seven studies reported results of objective educational assessments [21, 23, 27, 32, 36–38]. Two studies used structured interviews [31, 36].

### How has WhatsApp® been used as a learning tool in medical education?

Sixteen studies (69.6%) used WhatsApp® groups solely for educational purposes with a learning period from 2 days to 2 years (median duration 20 weeks).[17–38] All but one of these groups were moderated by a facilitator and most (13/16; 81.3%) were conducted in a local university or hospital setting. Seven used WhatsApp® in a blending learning setting, combining it with non-eLearning strategies [17, 27, 31, 34–37]. Only five of these sixteen studies [23, 27, 30, 36, 37] articulated a pre-defined syllabus for WhatsApp® learning, most relying on ad hoc recent clinical cases to drive online discussions.

The seven remaining studies described WhatsApp® groups that included non-educational discourse [10, 20, 22, 25, 28, 29, 32]. This included sharing the clinical aspects of patient care, organisational and scheduling information, emotional support and social messages. Only one of these studies had a designated moderator [29] and a majority (4/7; 57.1%) occurred at a national or international level.

### How has WhatsApp® been evaluated as a learning tool in medical education?

We grouped the methods of evaluating WhatsApp® into three categories; technical/logistical aspects of the medium; learner/learning activity during discussions; and educational outcomes of WhatsApp® interventions.

#### Technical/logistical aspects of the medium

Twelve articles reported data on the technical/logistical aspects of WhatsApp®, mostly drawn from user surveys [10, 17, 22, 23, 25, 26, 31, 33–36, 38]. The most cited benefit of WhatsApp® was its ability to create new learning opportunities, when geographical or time constraints meant that “meeting face-to-face is not possible” (p569) [25], described as “anytime, anywhere learning” [34, 38]. Access to learning material outside working hours was an advantage [17, 38] but also a factor contributing to WhatsApp®‘s intrusiveness [24, 31, 33, 35] with “message flooding” [23] and “WhatsApp® overload” [25].

Technical disadvantages cited were the necessity for internet access and compatible hardware devices, and poor image quality [17, 26, 34]. Technical advantages over other social media platforms (e.g. Facebook®) included easier image upload, quicker access and message posting, and the low cost and ease of use [23, 26, 31]. Several studies noted the high investment required by faculty to maintain the group discussions [24, 34–36, 38] and to prevent learner disengagement over time [30, 36].

#### Learner/learning activity during WhatsApp® discussions

Twelve studies analysed the content of WhatsApp® group discussions [10, 18, 19, 21–24, 28, 29, 31, 33, 36]. A common theme was the use of multimedia – visual and audiovisual tools – to promote discussion and learning [18, 19, 23, 24, 33]. These included ECGs, [18, 33] infectious disease files [23, 37], histopathology slides [19, 24, 38], dermatology images [26], and anatomy images [34]. A second group of studies stimulated learning mainly through textual engagement; asking questions, posing problems, and moderating learner discussions [21, 23, 31]. A third group mainly used the online space for information sharing, much of which was non-educational in nature [10, 22, 28, 29]. Two aspects of WhatsApp® discussions – passive participants and social discussion – were perceived to impede learning [10, 22–24, 29, 31, 36, 38].

#### Educational outcomes of the medium

A majority of studies (*n* = 13; 56.5%) reported only Kirkpatrick 1 learning outcomes [10, 17, 19, 20, 24–26, 28, 29, 31, 33–35]. These are summarised in Table 2. Eight studies reported level 2 outcomes [21, 23, 27, 30, 32, 36–38], one of which also reported a level 3 outcome [21]. The remaining 2 studies reported no Kirkpatrick outcomes [18, 22].

**Table 2:** Kirkpatrick level 1 learning outcomes from studies included in the scoping review

Positive	
Convenient and efficient method of learning and solving difficult clinical problems [10, 19, 23, 29, 32]	
Enables learning by numerous means;	
- By revision [17, 31]	
- By Q&A problem solving strategy [17]	
- By preplanned curriculum [23, 30] or by adapting to an evolving curriculum [31]	
- By using multimedia tools to explain complex concepts [31]	
- By teacher-learner and learner-learner model [23]	
- By learning in a legitimate, collaborative, social, online group space [23, 25, 31]	
- By deconstructing hierarchy, reducing inhibitions and encouraging active involvement by all grades of learner [21, 26, 31]	
- By obtaining links to relevant learning material [10, 23, 26]	
Enables assessment;	
- Formative assessment within discussions [21, 24, 30]	
- Summative assessment tool, especially as a method for measuring learner engagement/participation in discussions [17, 31]	
Negative	
Intrusiveness and interference with routine clinical work [24, 31]	
Large volume of learning material can impede learning [23, 25]	
Concerns about breaching patient confidentiality [24, 26]	
Effective learning depends on “completion” of a discussion topic which does not always happen [26]	

Seven studies assessing a change in knowledge reported a benefit from WhatsApp® discussions but each study had flaws limiting its conclusions. Three used a pre−/post-intervention assessment tool and showed an improvement in learner knowledge but did not include a control group [21, 36, 38]. The remaining four studies had a control group, comparing blended learning using WhatsApp® with traditional teaching. Of these, three studies demonstrated improved knowledge in the WhatsApp® groups but omitted baseline pre-intervention testing [27, 32, 37]. The final study compared 2 months of WhatsApp® learning with didactic lectures, using a control group and pre−/post-intervention testing [23]. Significant improvements in learner knowledge were reported in each group but not between groups.

### What educational theoretical principles were evident in studies of WhatsApp® as a learning tool in medical education?

Five of the twenty-three studies articulated a theoretical basis for learning – either eLearning theory [41] or mobile learning [42] – which guided the research design [23, 27, 31, 34, 35]. Two studies used their findings to subsequently suggest a theory informing learning in WhatsApp® groups; andragogy [34, 43] and peer-assisted learning [28]. Notwithstanding, there was indirect evidence of technology-rich orientations throughout many of the other studies, in particular cognitive theory of multimedia learning [44] and Harasim’s theory of online collaboration [45].

Some non-technological theories also bridged numerous studies. Several studies identified the importance of group learning in WhatsApp® users [18, 22–25, 28, 31, 32], reflecting influences such as an online community of practice [46], and social learning theory [47]. Motivational theory was also evident, in particular the ARCS model [48], whereby the convenience of WhatsApp® facilitated learner attention, the subject matter was relevant, learners were confident in the non-hierarchical environment and learner satisfaction was apparent in several of the studies’ results [10, 21, 31, 32].

Cognitive load theory [49] was relevant to studies where the high volume of learning material was thought to impede learning [23–25]. The user-friendly, familiar platform minimised extraneous cognitive load, prioritising the germane load of the online learning activities. Constructivism was a key theoretical construct in studies demonstrating learning built upon learners’ contributions rather than on student-facilitator dynamics [10, 18, 22, 28, 31].

## Discussion

In reviewing published literature on the role of WhatsApp® in medical education, we have shown that, in line with its widespread use as an instant messaging tool, WhatsApp® has been evaluated in numerous subspecialties in both undergraduate and postgraduate settings. Notwithstanding the design decisions, the risks of bias and scant theoretical foundations, a total of sixteen studies described its use primarily for educational purposes, of which seven reported, in a total of 647 learners, an improvement in learner knowledge, and one reported a change in learner behaviour. Therefore, while our findings highlight the convenience, efficiency, versatility and popularity of WhatsApp®, they also suggest that it may be an effective educational tool. The main finding of our review however is that there is a need for well-designed rigorous educational research with strong theoretical foundations to more clearly define the role and benefits of learning with an IMA.

Does it matter that an online platform such as WhatsApp® – a social phenomenon that is cheap and popular – is of any real educational benefit? Perhaps the answer depends on the purpose for which a WhatsApp® group discussion is designed. Medical educators should ideally use learning resources and instructional design principles which have a theoretical basis and have demonstrable learning benefits. Conversely, health professionals reaching out to other like-minded colleagues and peers to share clinical and learning resources, in a local, national or international setting are not bound by such rigorous educational standards; current evidence strongly suggests that WhatsApp® is a suitable resource for their purposes and that further research in this area is not warranted.

Although all of the included articles used WhatsApp® in a similar manner, of more importance were the individual study design decisions about how instant messaging could drive learning. In some studies, WhatsApp® provided an online space for healthcare staff to share experiences, opinions and resources [10, 22, 25, 28, 29], and to offer professional or emotional support to like-minded participants. These groups did not have a primary educational agenda, though educational elements were perceived throughout the discussions. Dedicated facilitators were not used, groups usually had national or international representation, all enrolled postgraduate users, and the duration of discussions were long, usually beyond 1 year. Educational assessment was limited to user attitudes.

Five other studies used WhatsApp® as a primary education tool with a pre-defined learning curriculum [23, 27, 30, 36, 37]. All groups had a dedicated faculty moderator, had a finite duration (2 days to 5 months), were mainly (4/5; 80%) in a local institutional setting and for undergraduate (4/5; 80%) learners. All five studies assessed Kirkpatrick level 2 outcomes, and notwithstanding some methodological flaws, all showed an improvement in learner knowledge or confidence following WhatsApp® learning.

Between these 2 groups were eleven studies using WhatsApp® as an educational tool but without a formal learning curriculum. In these studies, WhatsApp® discussion occurred on an ongoing basis (up to 2 years), with impromptu learning opportunities, stimulated by available clinical cases. Most (7/11; 63.6%) were in a postgraduate setting and most (9/11; 81.2%) were within a local institution or department. Most of these studies (9/11; 81.2%) assessed only learner attitudes, perhaps reflecting the flexible and ad hoc nature of this learning strategy.

**Fig. 2:**
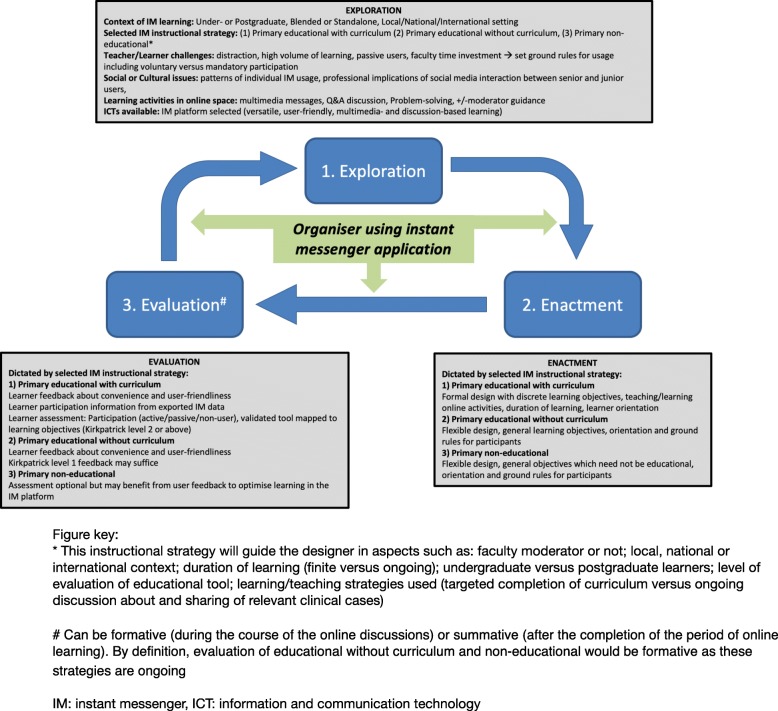
Proposed design model of instant messenger learning in medical education. Adapted from Dabbagh [50]

The objectives of these three strategies are quite different; a safe online space for postgraduate peer discussions; discrete learning modules designed around the IMA; a continuous online learning environment driven by topical clinical cases. Guided by these 3 strategies, we propose a design model of IMA learning, drawing from Dabbagh and Bannan-Ritland’s exploration-enactment-evaluation learning design framework for online education [50], in turn informed by socio-cultural and constructivist theories (Fig. 2) [51]. We propose that this stands distinct from less specific models of technology enhanced learning, eLearning or mobile learning. Our model may be a useful resource for educators and/or healthcare professionals planning to use an IMA in their practice. It may also help to fill the theoretical vacuum apparent in many of the educational studies reported in our review, addressing the truism that well-designed educational research should have a strong learning theory foundation [52].

Our findings add to existing literature in this field. In common with our findings, a recent review of 29 studies evaluating social media in graduate medical education identified a majority of descriptive studies with pre−/post-intervention assessment, Kirkpatrick level 1 and 2 outcomes, and “institutional-specific surveys” [14]. Their search however did not include studies evaluating WhatsApp®. A further review [13] of social media in undergraduate and postgraduate medical education identified 13 articles evaluating Facebook®, YouTube® and Twitter®, but not WhatsApp®. SM use showed “no correlation with student performance” (p374) and studies lacked “rigorous programmatic evaluation” (p374). In a review of the educational impact of Facebook®, Pander and Pinilla noted, in 16 studies, a preference for ongoing local learning rather than for curriculum-driven activities and “no conclusive evidence on the impact of the use of Facebook … on higher clinical competency levels and on patient-outcomes” [53] (p7). A very recent systematic review evaluating mobile hand-held devices for health professions described social media learning as an “unusual example of mobile devices supporting learning” [1] (p132). Our study therefore echoes and complements the findings of previous related literature, while strengthening the case for using IMAs in medical education and advancing a design theory for instant messenger learning.

Our review has potential limitations. It is possible that we have omitted relevant publications. Notwithstanding this, our sensitive search term, independent author searching, the updated second search strategy and the large number of identified articles when compared with other related reviews [54, 55] suggest a comprehensive coverage in our search results. Our conclusions and inferences are drawn from a heterogenous group of educational studies with inherent design flaws and with limited theoretical bases. This raises concerns about the generalisability and credibility of the included quantitative and qualitative data respectively. Nonetheless, our findings suggest there is mounting evidence supporting the use of IMAs in medical education. Our proposed design model may help medical educators adopt a more formal approach to incorporating IMAs into their daily practice.

## Conclusion

In conclusion, our review of WhatsApp® brings into focus the educational benefits of instant messaging and the strategies that have been used to employ this system in the medical setting. Our findings and the accompanying design model may provide a theoretical and practical framework for those planning to use IMAs in their educational practice. Well-designed research is warranted to further evaluate the role of IMAs in medical education but also to explore the utility of our design model to improve practice in this area.

## Data Availability

The dataset supporting the conclusions of this article is included within this article and its additional files.

## Abbreviations

IMA: Instant messenger applications
MID: Mobile internet device
mLearning: Mobile learning
SM: Social media

## References

[1] Maudsley G, Taylor D, Allam O, Garner J, Calinici T, Linkman K (2019). A best evidence medical education (BEME) systematic review of: what works best for health professions students using mobile (hand-held) devices for educational support on clinical placements? BEME guide no. 52. Med Teach.

[2] Chase TJG, Julius A, Chandan JS, Powell E, Hall CS, Phillips BL, Burnett R, Gill D, Fernando B (2018). Mobile learning in medicine: an evaluation of attitudes and behaviours of medical students. BMC Med Educ.

[3] Boruff JT, Storie D (2014). Mobile devices in medicine: a survey of how medical students, residents, and faculty use smartphones and other mobile devices to find information. J Med Libr Assoc.

[4] Bullock A, Webb K (2015). Technology in postgraduate medical education: a dynamic influence on learning?. Postgrad Med J.

[5] AlFaris E, Irfan F, Ponnamperuma G, Jamal A, Van der Vleuten C, Al Maflehi N, Al-Qeas S, Alenezi A, Alrowaished M, Alsalman R, Ahmed AMA (2018). The pattern of social media use and its association with academic performance among medical students. Med Teach.

[6] Tang Y, Hew KF (2017). Is mobile instant messaging (MIM) useful in education? Examining its technological, pedagogical, and social affordances. Educational Research Review.

[7] Garcia-Cabot A, de-Marcos L, Garcia-Lopez G (2015). An empirical study on m-learning adaptation: learning performance and learning contexts. Comput Educ.

[8] WhatsApp. WhatsApp [Internet]. About WhatsApp. [cited 2019 Apr 7]. Available from: https://www.whatsapp.com/.

[9] SimilarWeb. www.similarweb.com [Internet]. WhatsApp. [cited 2019 Apr 7]. Available from: https://www.similarweb.com/website/whatsapp.com.

[10] Carmona S, Alayed N, Al-Ibrahim A, D’Souza R (2018). Realizing the potential of real-time clinical collaboration in maternal–fetal and obstetric medicine through WhatsApp. Obstet Med.

[11] O'Sullivan E, Cutts E, Kavikondala S, Salcedo A, D'Souza K, Hernandez-Torre M, Anderson C, Tiwari A, Ho K, Last J (2017). Social media in health science education: an international survey. JMIR Med Educ.

[12] D’Souza K, Henningham L, Zou R, Huang J, O’Sullivan E, Last J, Ho K (2017). Attitudes of health professional educators toward the use of social media as a teaching tool: global cross-sectional study. JMIR Med Educ.

[13] Sutherland S, Jalali A (2017). Social media as an open-learning resource in medical education: current perspectives. Adv Med Educ Pract.

[14] Sterling M, Leung P, Wright D, Bishop TF (2017). The use of social media in graduate medical education: a systematic review. Acad Med.

[15] Arksey H, O’Malley L (2005). Scoping studies: towards a methodological framework. Int J Soc Res Methodol.

[16] Levac D, Colquhoun H, O’Brien KK (2010). Scoping studies: advancing the methodology. Implement Sci.

[17] Mayer G, Nicolson P, Morton S (2017). Transfusion education: can using social media help improve training? The West Midlands experience. Br J Haematol.

[18] Bhesania S, Chirumamilla S, Peterson S, Raza AS, Faroqui M, Mehta P (2018). Using social media to advance medical education in a university affiliated community residency program. J Gen Intern Med.

[19] Elshaikh A, Saeed O, El Hag MI (2018). Whatsapp as a supplemental learning tool for pathology. Lab Investig.

[20] Alkhalaf AM, Tekian A, Park YS (2018). The impact of WhatsApp use on academic achievement among Saudi medical students. Med Teach.

[21] Bakshi SG, Bhawalkar P (2017). Role of WhatsApp-based discussions in improving residents’ knowledge of post-operative pain management: a pilot study. Korean J Anesthesiol.

[22] Blumenfeld O, Brand R (2016). Real time medical learning using the WhatsApp cellular network: a cross sectional study following the experience of a division’s medical officers in the Israel defense forces. Disaster and Mil Med.

[23] Gon S, Rawekar A (2017). Effectivity of E-learning through Whatsapp as a teaching learning tool. MVP J Med Sci.

[24] Goyal A, Tanveer N, Sharma P (2017). WhatsApp for teaching pathology postgraduates: a pilot study. J Pathol Inform.

[25] Hayward E, Ward A (2018). Virtual learning communities for faculty members: does WhatsApp work?. Med Educ.

[26] Kaliyadan F, Ashique KKT, Jagadeesan S, Krishna B (2016). What’s up dermatology? A pilot survey of the use of WhatsApp in dermatology practice and case discussion among members of WhatsApp dermatology groups. Indian J Dermatology, Venereol Leprol.

[27] Khan AA, Siddiqui AZ, Mohsin SF, Al Momani MM, Mirza EH (2017). Impact of network aided platforms as educational tools on academic performance and attitude of pharmacology students. Pak J Med Sci.

[28] Loo JL, Koh EBY, Pang NTP, Nor Hadi NM. Use of WhatsApp in assisting psychiatry learning. 2016;50(11):1165. 10.1111/medu.13195 27762032

[29] Mazzuoccolo LD, Esposito MN, Luna PC, Seiref S, Dominguez M, Echeverria CM (2019). WhatsApp: a real-time tool to reduce the knowledge gap and share the best clinical practices in psoriasis. Telemed J E Health.

[30] Bukhari M, Morton AR, Benjamin KAT, Shamseddin KM (2017). Enhancing internal medicine trainees’ nephrology competency: Queen’s enphrology E-learning using WhatsApp (Q-new) study. Intern Med.

[31] Raiman L, Antbring R, Mahmood A (2017). WhatsApp messenger as a tool to supplement medical education. BMC Med Educ.

[32] Khanna V, Sambandam SN, Gul A, Mounasamy V (2015). “WhatsApp”ening in orthopedic care: a concise report from a 300-bedded tertiary care teaching center. Eur J Orthop Surg Traumatol.

[33] Kochar A, Rymer J, Samad Z, Banks A, Mandawat A, Sun A (2018). Disrupting fellow education through group texting: WhatsApp in fellow education?. J Am Coll Cardiol.

[34] Ranjan R, Jain A, Baghel AS (2017). WhatsApp-assisted learning of anatomy as an adjuvant to traditional classroom learning: achievements and prospect. Int J Anat Res.

[35] Mohesh G, Meerasa SS (2016). Perceptions on M-learning through WhatsApp application. J Educ Technol Health Sci.

[36] Dyavarishetty PV, Patil DC (2017). An interventional study to assess the effectiveness of ‘WhatsApp’ as a teaching learning tool in community medicine. Int J Community Med Public Health.

[37] Mohanakrishnan K, Jayakumar N, Kasthuri A, Nasimuddin S, Malaiyan J, Sumathi G (2017). Whatsapp enhances medical education: is it the future?. Int J Med Sci Public Health.

[38] Maske SS, Kamble PH, Kataria SK, Raichandani L, Dhankar R (2018). Feasibility, effectiveness, and students’ attitude toward using WhatsApp in histology teaching and learning. J Educ Health Promot.

[39] Centre for Evidence-Based Medicine. Study Designs - CEBM [Internet]. [cited 2019 Apr 23]. Available from: https://www.cebm.net/2014/04/study-designs/.

[40] Kirkpatrick D (1998). Evaluating Training Programs.

[41] Clark RC, Mayer RE (2008). eLearning and the science of instruction. 2nd Ed.

[42] Crompton H, Berge ZL, Muilenburg LY (2013). A historical overview of M-learning: toward learner-centred education. Handbook of mobile learning.

[43] Knowles MS, Holton EF, Swanson RA (2015). The adult learner. 8th Ed.

[44] Mayer RE, Mayer RE (2005). Cognitive theory of multimedia learning. The Cambridge handbook of multimedia learning.

[45] Harasim L (2012). Learning theories and online technologies.

[46] Wenger E (1998). Communities of practice; learning, meaning and identity.

[47] Bandura A (1977). Social learning theory.

[48] Keller JM (2010). Motivational design for learning and performance.

[49] Sweller J, Ayres P, Kalyuga S (2011). Cognitive load theory.

[50] Dabbagh N, Bannan-Ritland B (2005). Online learning: concepts, strategies and application.

[51] Dabbagh N (2005). Pedagogical models for e-learning: a theory-based design framework. International Journal of Technology in Teaching and Learning.

[52] O’Connor E, Moore M, Cullen W, Cantillon P (2017). A qualitative study of undergraduate clerkships in the intensive care unit: It’s a brand new world. Perspect Med Educ.

[53] Pander T, Pinilla S, Dimitriadis K, Fischer MR (2014). The use of Facebook in medical education - a literature review. GMS Z Med Ausbild.

[54] Giordano V, Koch H, Godoy-Santos A, Belangero WD, Santos Pires RE, Labronici P (2017). WhatsApp messenger as an adjunctive tool for telemedicine: an overview. Interact J Med Res.

[55] Dunleavy G, Nikolaou CK, Nifakos S, Atun R, Law GCY, Tudor Car L (2019). Mobile digital education for health professionals: systematic review and meta-analysis by the digital health education collaboration. J Med Internet Res.

